# Haptoglobin (*HP*) and Haptoglobin-related protein (*HPR*) copy number variation, natural selection, and trypanosomiasis

**DOI:** 10.1007/s00439-013-1352-x

**Published:** 2013-09-05

**Authors:** Robert J. Hardwick, Anne Ménard, Manuela Sironi, Jacqueline Milet, André Garcia, Claude Sese, Fengtang Yang, Beiyuan Fu, David Courtin, Edward J. Hollox

**Affiliations:** 1 Department of Genetics, University of Leicester, Leicester, UK; 2 Scientific Institute IRCCS E. Medea, Bioinformatic Lab, 23842 Bosisio Parini, Italy; 3 Institut de Recherche pour le Développement (IRD), UMR 216 Mère et enfant face aux infections tropicales, Centre d’Etude et de Recherche sur le Paludisme Associé à la Grossesse et à l’Enfance (CERPAGE); Faculté des Sciences de la Santé, Cotonou, Benin; 4 IRD, UMR 216 Mère et enfant face aux infections tropicales, Université Paris Descartes, Paris, France; 5 Faculté de Pharmacie, Université Paris Descartes, Sorbonne Paris Cité, France; 6 Programme National de lutte contre la trypanosomose humaine africaine (PNLTHA), Kinshasa, Democratic Republic of Congo; 7 Wellcome Trust Sanger Institute, Hinxton, UK

## Abstract

Haptoglobin, coded by the *HP* gene, is a plasma protein that acts as a scavenger for free heme, and haptoglobin-related protein (coded by the *HPR* gene) forms part of the trypanolytic factor TLF-1, together with apolipoprotein L1 (ApoL1). We analyse the polymorphic small intragenic duplication of the *HP* gene, with alleles Hp1 and Hp2, in 52 populations, and find no evidence for natural selection either from extended haplotype analysis or from correlation with pathogen richness matrices. Using fiber-FISH, the paralog ratio test, and array-CGH data, we also confirm that the *HPR* gene is copy number variable, with duplication of the whole *HPR* gene at polymorphic frequencies in west and central Africa, up to an allele frequency of 15 %. The geographical distribution of the *HPR* duplication allele overlaps the region where the pathogen causing chronic human African trypanosomiasis, *Trypanosoma brucei gambiense*, is endemic. The *HPR* duplication has occurred on one SNP haplotype, but there is no strong evidence of extended homozygosity, a characteristic of recent natural selection. The *HPR* duplication shows a slight, non-significant undertransmission to human African trypanosomiasis-affected children of unaffected parents in the Democratic Republic of Congo. However, taken together with alleles of *APOL1*, there is an overall significant undertransmission of putative protective alleles to human African trypanosomiasis-affected children.

**Electronic supplementary material:**

The online version of this article (doi:10.1007/s00439-013-1352-x) contains supplementary material, which is available to authorized users.

## Introduction

Haptoglobin (Hp), encoded by the gene *HP*, is an abundant acute-phase glycoprotein in the plasma which binds free haemoglobin (Hb) that has been released by lysis of erythrocytes, often as a result of infection. The resulting haptoglobin-haemoglobin complex is cleared by binding to the macrophage scavenging receptor CD163, followed by endocytosis. This process prevents oxidative damage and disruption to nitrous oxide homeostasis caused by free heme molecules (Nielsen and Moestrup 2009).

Because of its abundance in blood plasma, Hp was one of the first blood serum proteins to be analysed by native protein electrophoresis to identify polymorphic variation (Smithies 1955). Two electrophoretic alleles, termed Hp1 and Hp2, were subsequently characterised as resulting from a 1.7 kb intragenic duplication so that the Hp2 allele encodes a longer peptide chain than the Hp1 allele (Maeda et al. 1984; Smithies et al. 1962). The two alleles encode proteins that are functionally different, and have been associated with a variety of clinical conditions (Langlois and Delanghe 1996). There is evidence that homozygotes for the Hp2 allele are more protected against severe malaria (Atkinson et al. 2007; Quaye et al. 2000), although such a link remains controversial (Aucan et al. 2002; Bienzle et al. 2005).

**Fig. 1:**
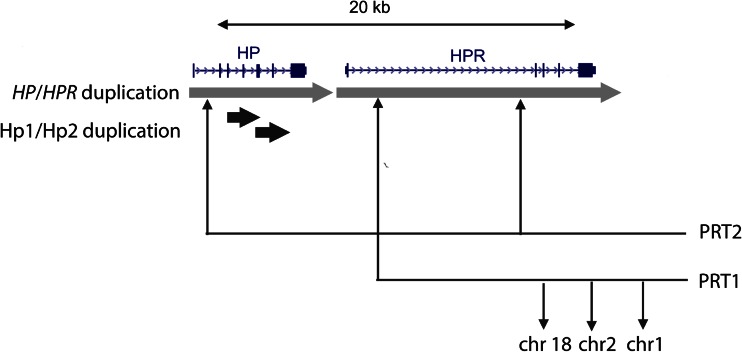
Segmental duplications of *HP* and *HPR.* The *HP*/*HPR* duplication is shown in *light grey*, with a percentage identity of ~94 %, apart from a 9.3 kb LTR insertion which distinguishes the *HPR* copy, and is responsible for the difference in duplication size. The small 1.6 kb duplication responsible for the Hp2 (duplicated) and Hp1 (not duplicated) alleles is shown in black, with a percentage identity of 98 %. Locations of loci amplified for the two paralog ratio tests (PRT) used are also shown

The entire *HP* gene is within a tandemly-repeated 16 kb segmental duplication on chromosome 16, with the other duplicated copy sharing 94 % DNA sequence identity and containing the *HPR* gene which encodes haptoglobin-related protein (Hpr) (Fig. 1). Copy number variation of the *HPR* gene has been reported in African-Americans, where extra tandemly-arranged copies of the *HPR* gene have been generated by non-allelic homologous recombination (NAHR) (Maeda et al. 1986). Using pulsed-field gel electrophoresis and Southern blots, the study identified 5 individuals carrying additional copies of *HPR* in a sample of 15 individuals, with quantification of Southern blot bands suggesting that some individuals had up to 5 copies of the *HPR* gene. The authors speculated whether such increase in gene copy number could be adaptive, and recent studies on other copy number variations (CNVs) have suggested an adaptive role for copy number variation in response to pathogen pressure and other environmental changes (Iskow et al. 2012).

Like Hp, Hpr binds free heme with high-affinity, but the resulting Hpr-Hb complex does not bind to CD163; instead it persists in the serum bound to apolipoprotein L1 (ApoL1) (Nielsen et al. 2006). Hpr protein has an important role in protection against *Trypanosoma brucei*, the pathogen that causes human African trypanosomiasis, also known as sleeping sickness (Barrett et al. 2003; Smith et al. 1995). Trypanosomes rely on binding and internalisation of circulating plasma haptoglobin-haemoglobin (Hpr-Hb) to acquire iron necessary for their survival. Together with ApoL1, Hpr-Hb forms a protein complex called the trypanosome lytic factor-1 (TLF-1), which uses the trypanosome’s receptor for haptoglobin to deliver ApoL1 into the lysosomal compartment of the trypanosome, where the low pH triggers lysis (Drain et al. 2001; Vanhollebeke et al. 2008). This Trojan horse approach mediates effective killing of the trypanosome. TLF-1 causes effective lysis of *T. brucei brucei*, a zoonosis from cattle which infects humans but is self-resolving. However, *T. brucei rhodesiense,* which causes acute human African trypanosomiasis (HAT) in East Africa, is protected against TLF-1 by the parasite’s SRA gene. In addition, TLF-1 does not appear to be effective in vitro against *T. brucei gambiense*, which is currently endemic to West and Central Africa, causes chronic HAT, and is responsible for most deaths from this disease. This is due, at least in part, to coding sequence changes in *T. brucei gambiense* that reduce the affinity of the receptor for TLF-1 (Kieft et al. 2010). Hpr is also a component of trypanosome lytic factor 2 (TLF-2), but this is less stable, less-studied and appears to contain many other components (Raper et al. 1999).

The evidence for interaction of *HP* and *HPR* genes with different pathogens prompted us to explore the Hp1/Hp2 polymorphism and the CNV of the *HPR* gene in populations from around the world, investigate the role of selection on alleles of these polymorphisms, and test the role of increased *HPR* copy number in susceptibility to HAT.

## Methods

### DNA samples

952 DNA samples from 52 populations were obtained from the CEPH-Human Genome Diversity Project (HGDP) (Cann et al. 2002; Rosenberg 2006). DNA samples used in the HapMap Phase 1 project (CEU, European Americans from Utah; YRI, Yoruba from Ibadan, Nigeria; CHB, Chinese from Beijing; JPT, Japanese from Tokyo) were obtained from Coriell Cell Repositories.

The Yansi samples from the Democratic Republic of Congo (DRC) and HAT phenotyping have been fully described previously (Courtin et al. 2007). Positive cases were identified if both serology (card agglutination test) and parasitology (direct microscopic examination of blood or lymph for parasites) were positive. DNA from 353 individuals was collected, comprising of 135 cases and 218 related controls, consisting of 109 pedigrees. All individuals were born in the area and exposed to the risk of infection since birth. The study was approved both by the ethics committee of the DRC Public Health Ministry and local traditional authorities.

### Hp1/2 genotyping

Genotyping the Hp1/2 polymorphism was performed using a PCR approach, developed previously (Koch et al. 2002). Briefly, the assay consists of two separate PCR reactions that generate PCR products of characteristic size dependent of the genotype, which then can be separated by agarose gel electrophoresis and visualised by ethidium bromide staining (supplementary figure 1). Primers A and B (supplementary table 1) amplify a 1,757 bp region from the Hp1 allele and a 3481 bp region from the Hp2 allele. To control for the possibility of the longer product being absent because of highly sheared genomic DNA, primers C and D amplify the junction fragment specific to the Hp2 duplicated allele, generating a 349 bp product. Seven control DNAs (supplementary table 2) with different genotypes were included with every experiment.

### Copy number typing using the paralog ratio test

Two paralog ratio tests (PRTs) (Armour et al. 2007) were designed to measure *HPR* copy number, by identifying paralogous segments of the haptoglobin region using the BLAST-like Alignment Tool (Fig. 1). PRT1 assumes *HP* itself is not copy number variable. Deletion of *HP* has been observed as a cause of anhaptoglobinemia in Asians, with a frequency of <3 % (Koda et al. 1998). However, we did not see any evidence of this allele that we predict would generate a clear discrepancy between results from PRT1 and PRT2. The second PRT, using primers HP_PRT_1F and HP_PRT_1R (supplementary table 2), amplifies *HPR* and not *HP*, because it is targeted to the LTR insertion in the *HPR* intron, and co-amplifies several reference regions on other chromosomes, providing a second independent measure of *HPR* copy number. Both PRTs were performed together as a duplex PCR, in 1× Kapa PCR Buffer A (1.5 mM final Mg^2+^ concentration), 0.5u *Taq* DNA polymerase (Kapa Biosystems), 3 pmol of each primer and 5–10 ng genomic DNA in a final volume of 10 μl. PCR cycling conditions were 98 °C for 2 min, followed by 23 cycles of 98 °C for 20 s, 57 °C for 30 s and 70 °C for 1 min, followed by a final extension step of 70 °C for 10 min. 2 μl of the PCR product was added to 10 μl formamide with MapMarker400 ROX-labelled size standard (Eurogentec), denatured at 96 °C for 3 min, and then electrophoresed on an ABI3130XL capillary electrophoresis machine.

Quantification of peaks of the electropherogram was performed using GeneMapper (Applied Biosystems), with samples rerun if peak signal was saturated or very weak. Copy number of *HPR* was estimated by firstly calculating the ratio of test: reference peak area for both PRTs, and correcting for inter-experimental variation by calibrating the ratio against the ratios of seven known copy number controls, included in each experiment (supplementary table 2). The distribution of the average corrected ratios of the two PRT values for each sample, including all controls and replicates, was fitted to a Gaussian mixture model using the CNVtools package (Barnes et al. 2008), implemented in the statistical language R. Following the removal of three samples (HGDP640, NA18503_13, NA19221_13) as outliers, three Gaussian curves were fitted, constraining the means to be proportional to copy number and the variance of each distribution to be the same, reflecting distributions of samples for *HPR* copy numbers of 2, 3 and 4. These Gaussian curves were used to generate an integer copy number call of 2, 3 or 4 for each sample, together with a posterior probability for each call.

### *APOL1* genotyping

The three variant sites analysed were two SNPs (rs73885319 and rs60910145) and one 6 bp-indel (rs71785313). These were amplified together in a single PCR product, using standard PCR conditions and primers APOL1F and APOL1R (supplementary table 1). The alleles at rs73885319 were distinguished by *Hind*III restriction enzyme (A-cut, G-uncut), alleles at rs60910145 distinguished by *Nla*III restriction enzyme (G-cut, T-uncut) and alleles at rs71785313 by the 6 bp size difference following capillary electrophoresis on an ABI3130xl.

### Fiber Fluorescent in situ Hybridisation (Fiber-FISH)

Fiber-FISH was performed as described previously (Perry et al. 2008). Briefly, stretched DNA fibers were prepared from lymphoblastoid cell lines. A fosmid clone (G248P85613E6) that contains the *HPR* gene and a reference clone (G248P84443C9) was obtained from the clone archive resource of the Wellcome Trust Sanger Institute. Fosmid DNA was prepared using the Phase-Prep BAC DNA kit (Sigma-Aldrich) following the manufacturer’s protocol. The *HPR* clone was labelled with Dinitrophenol (DNP)-11-dUTP (PerkinElmer) and detected with rabbit anti-DNP and Alexa 488 conjugated goat anti-rabbit IgG. The reference clone was labelled with Digoxigenin (DIG)-11-dUTP (Roche) and detected with monoclonal mouse anti-DIG IgG (Sigma-Aldrich) and Texas red conjugated donkey anti-mouse IgG (Invitrogen). After detection, slides were mounted with SlowFade Gold^®^ (Invitrogen) mounting solution containing 4′,6-diamidino-2-phenylindole (Invitrogen). Images were captured on a Zeiss Axioplan fluorescent microscope and processed with the SmartCapture software (Digital Scientific UK).

### Population genetic analyses

*F*_ST_ calculations were performed using Arlequin 3.5 or the R package HIERFSTAT (Excoffier and Lischer 2010; Goudet 2004). For each pair of populations, the percentile rank of *F*_ST_ for the *HPR* duplication was obtained by comparison with the distribution of *F*_ST_ values calculated for SNPs genotyped in the HGDP panel and showing a similar minor allele frequency (MAF) as the *HPR* allele. Specifically, for each pairwise comparison the mean MAF of the *HPR* duplication in the two populations was calculated and HGDP SNPs in a MAF range of ±0.02 were used to obtain the distribution of *F*_ST_ values.

Pathogen absence/presence matrices were constructed for the 21 countries where the HGDP populations are located, based on the Gideon database, as described previously (Fumagalli et al. 2009). Briefly, pathogen diversity was calculated from these data for each population, taking into account only species/genera that are transmitted in the 21 countries, meaning that cases of transmission caused by tourism and immigration were not taken into account; also, species that have recently been eradicated as a result, for example, of vaccination campaigns, were recorded as present in the matrix. Malaria prevalence was obtained from either the Gideon or WHO databases, as previously described (Pozzoli et al. 2010). To account for the demographic history of human populations, correlations were calculated using partial Mantel tests. Specifically, matrices were computed as pairwise Euclidean distances in allele frequency, distance from East Africa, and pathogen diversity or malaria prevalence (either from the WHO or Gideon). Distances from Africa were derived from a previous work (Handley et al. 2007) and refer to a model of human migration from East Africa along landmasses and avoiding mountain regions with altitude over 2,000 m. The statistical significance of correlation tests was calculated by performing 10,000 permutations of pathogen diversity or malaria prevalence within continental regions; these were defined as previously suggested (Li et al. 2008) (i.e. Africa, Europe, America, Central-South Asia, East Asia, Oceania) with Middle Eastern populations grouped with Europeans. Partial Mantel correlations were performed using the Vegan R package.

### Haplotype phasing

Haplotype phasing was performed using the Bayesian method implemented in PHASE 2.1 (Stephens and Donnelly 2003). For short-range haplotype analysis, SNP genotypes of HGDP and HapMap samples for 8 SNPs flanking the *HP*/*HPR* CNV region were downloaded using the SPSmart portal (Jorge et al. 2008). These 8 SNPs spanned 55 kb immediately flanking the *HP*/*HPR* CNV region, and were selected on the basis of genotypes being available on the HGDP panel, and not being within the copy number variable region itself. The Hp1/2 polymorphism and the HPR duplication polymorphism were coded as diallelic SNPs for phasing. For long-range haplotype phasing, SNP genotypes from 2 Mb surrounding the *HP* gene for the YRI population were downloaded from the International HapMap Project (release 23a) and from the CEPH-HGDP website. The HapMap data consisted of 2218 genotypes (~1 SNP per kb) and HGDP data consisted of 394 genotypes (~1 SNP per 5 kb) from a custom Affymetrix SNP chip (Genome-wide Human Origins 1) courtesy of David Reich and colleagues. The design of this SNP chip was informed by low-coverage resequencing of 12 CEPH-HGDP samples and the low-coverage sequencing of the archaic hominids Neanderthal and Denisovan, and therefore the SNPs represented on the chip are likely to be more representative of common global genetic diversity. For the YRI, SNP genotypes with non-Mendelian inheritance were removed, and, for phasing using PHASE, all data were prepared using the software PLATO (http://ritchielab.psu.edu/ritchielab/project-plato/).

### Extended haplotype analysis

We used the R package REHH for all extended haplotype analyses and plots (Gautier and Vitalis 2012). SNP physical map positions were converted to genetic map positions based on the Rutgers second-generation linkage map (Matise et al. 2007). Extended haplotype homozygosity (EHH, Sabeti et al. 2002), was calculated for both Hp1/2 and *HPR* duplication polymorphisms, for all SNPs until EHH <0.05. The integrated haplotype score (iHS) was calculated on all SNPs, with an allele frequency bin of 0.2 to standardise iHS scores against other SNPs of its frequency class within the region. *P* values were calculated assuming a Gaussian distribution of iHS scores under the neutral model, this assumption was checked by plotting the values against a Gaussian distribution. Age of the *HPR* duplication was estimated from linkage disequilibrium using the equation EHH ≈ Pr(Homozygosity) = *e* ^−2rg^, where r is recombination rate in Morgans and g is the age in generations (Voight et al. 2006). Rearranging to give −ln(EHH) ≈ 2rg, we estimated the age of the allele by regressing the values of −ln(EHH) at various genetic distances 2r from the *HPR* allele, the gradient of the regression line being equal to g. Estimates of age in years were converted by multiplying the allele age by the generation time, estimated to be 27 years (Fenner 2005).

### Family-based association tests

Family-based association tests were performed for the *HPR* duplication and three SNPs in the *APOL1* gene using FBAT v2.0.4 software (De et al. 2013; Horvath et al. 2001). Single variant tests were performed under an additive model, and the empirical variance (the −e option) was used to ensure its validity as a test of association in the pedigree. Each variant was analysed in turn and together using a collapsing method, originally designed for rare variant analysis. The unweighted statistic was calculated (using option −v0), because of the similarity of minor allele frequency of each polymorphism, and the vulnerability of low minor allele frequencies to stochastic sampling variation in a small dataset.

## Results

### Accuracy of *HPR* copy number calling

Precise and accurate calling of copy number presents technical challenges. Where the copy number variable region is small and the structure well defined, PCR across the whole region followed by separation by size, and junction fragment PCR, are robust strategies that we use here to genotype the 1.7 kb duplication responsible for the Hp1/2 polymorphism. However, for larger CNV regions, often with unclear structures, quantification of DNA sequence by hybridisation or quantitative PCR strategies are often used, but such methods are prone to noise and need to be well-validated.

**Fig. 2:**
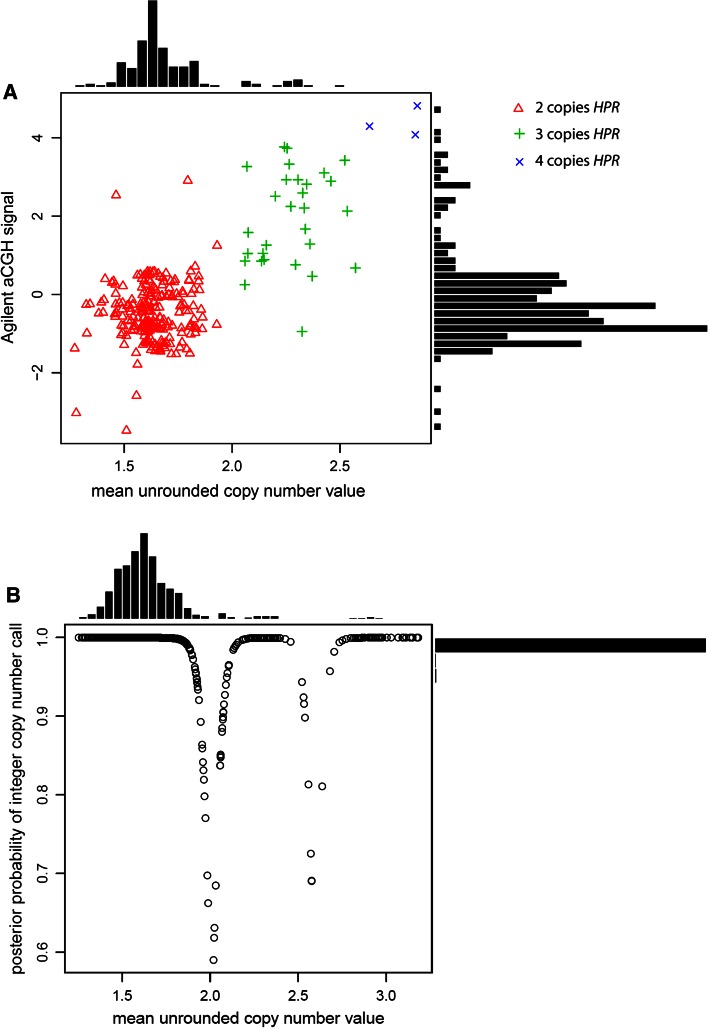
Assessment of *HPR* copy number assay quality. **a** Scatterplot and associated histograms showing mean unrounded copy number values generated by PRT1 and PRT2 (x-axis) plotted against array-CGH signal (y-axis) for the 270 HapMap Phase I DNA samples. Points are labelled according to the final diploid copy number inferred by Gaussian mixture modelling. **b** Scatterplot and associated histograms showing mean unrounded copy number values generated by PRT1 and PRT2 (x-axis) plotted against posterior probability of the final diploid copy number call, for HapMap and HGDP samples

Here, we use the paralog ratio test (PRT), a form of quantitative PCR, to measure *HPR* copy number (Armour et al. 2007; Walker et al. 2009). Initial inspection of array-CGH (aCGH) copy number calls for this region, generated by the Agilent CNV association chip, together with previous copy number calling on aCGH data, suggested that there were three genotype classes, with the two classes showing an increase in signal most likely reflecting heterozygous and homozygous duplications of the *HPR* gene (3 and 4 copies respectively) (Conrad et al. 2009). This allowed selection of seven HapMap samples of different copy number which would be used as positive controls in each experiment. We then typed the 270 HapMap samples, and, for each sample, compared our raw copy number estimate with the integer copy number calls and with the value of the first principal component of the 14 Agilent aCGH probes spanning the CNV data for this region (Fig. 2a). We also compared our raw copy number signal with the quality of integer copy number call (Fig. 2b). 95.6 % of all calls had a posterior probability of more than 0.98, 99.2 % of all calls had a posterior probability of more than 0.8.

**Fig. 3:**
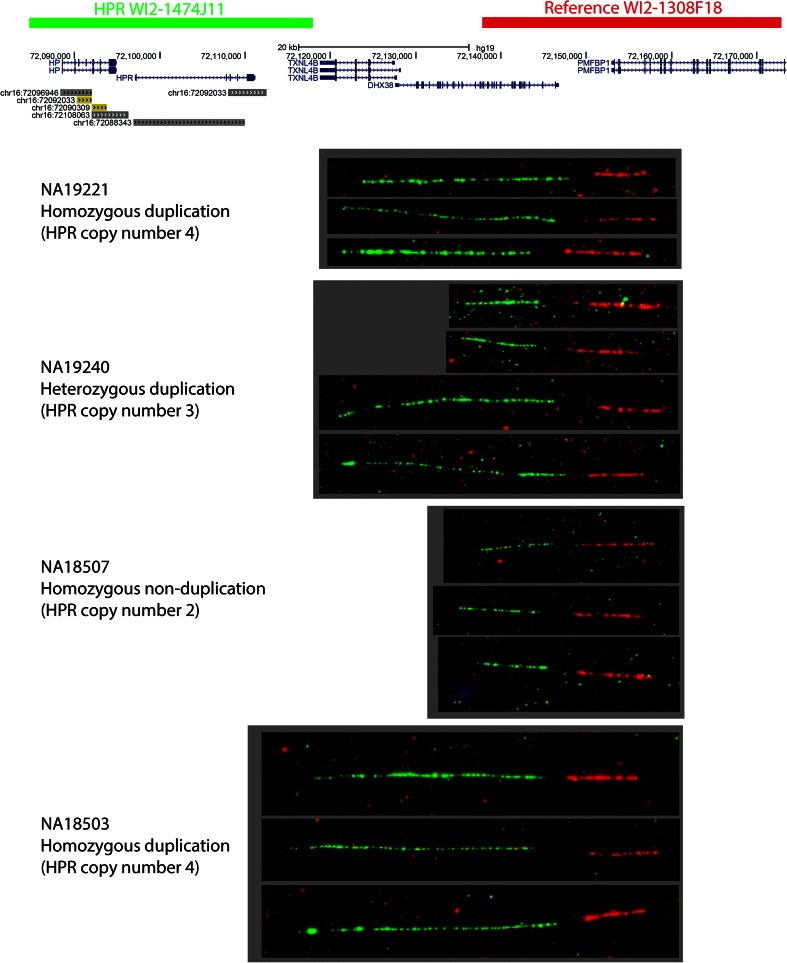
Visualisation of *HPR* duplication using fiberFISH. Two fosmid clones, corresponding to the regions shown at the top of the figure, were hybridised to stretched DNA fibers from lymphoblastoid cell lines of four HapMap samples. The *HPR* duplication is clearly shown as an increased length of green signal on the DNA fiber

As a further check of the *HPR* copy number of the control samples, four of their corresponding cell lines were used to generate DNA fibers for validation by fiber-FISH. A 38.7 kb reference fosmid distal to the CNV region (visualised in red), and a 39.4 kb test fosmid covering the CNV region (visualised in green) were hybridised to DNA fibers. Figure 3 shows example images from each experiment, clearly showing the extended length of green signal in both *HPR* duplication homozygotes, and both short and long green signals on fibers from the *HPR* duplication heterozygote. The ratio of the difference between the long and short green signals is between 2:1 and 3:1. This is consistent with duplication in tandem spanning the full length of the *HPR* gene, corresponding to the annotated segmental duplication containing the *HPR* gene. However, the ratio is longer than the 2:1 ratio expected, and could also be consistent with a triplication in tandem or involvement of a larger segment of DNA in the duplication, beyond the annotated *HPR* segmental duplication. A duplication is more likely than a triplication given the change in raw ratios from the two PRT assays used (approximately 1:1.5:2 for homozygote, heterozygote and homozygote duplication respectively) and this is consistently seen when the HPR duplication is inherited in trios. The alternative explanation, that extra genetic material is involved in the duplication is perhaps more likely. Indeed, for NA19240, the long green signal allele is on a Hp2 haplotype (the Hp2 allele being a small intragenic duplication), while the short allele is on a Hp1 haplotype, potentially exaggerating the length difference. The extra copies of the *HPR* gene are known to include the large retrovirus element in its intron (Maeda et al. 1986), although it is also possible that the allele seen by our fiber-FISH contains extra retroviral or other high-copy repeat material in the intron. We analysed the previously published high density tiling-path Nimblegen aCGH data across this region from NA19240 (Conrad et al. 2009), which is heterozygous for the *HPR* duplication. This indicates the duplication is around 25 kb (supplementary figure 2), consistent with pulsed-field gel electrophoresis results from earlier studies (Maeda et al. 1986), although this method cannot detect DNA sequences, such as extra retroviral elements, that are not present in the reference sequence.

### Allele frequency in different populations

**Fig. 4:**
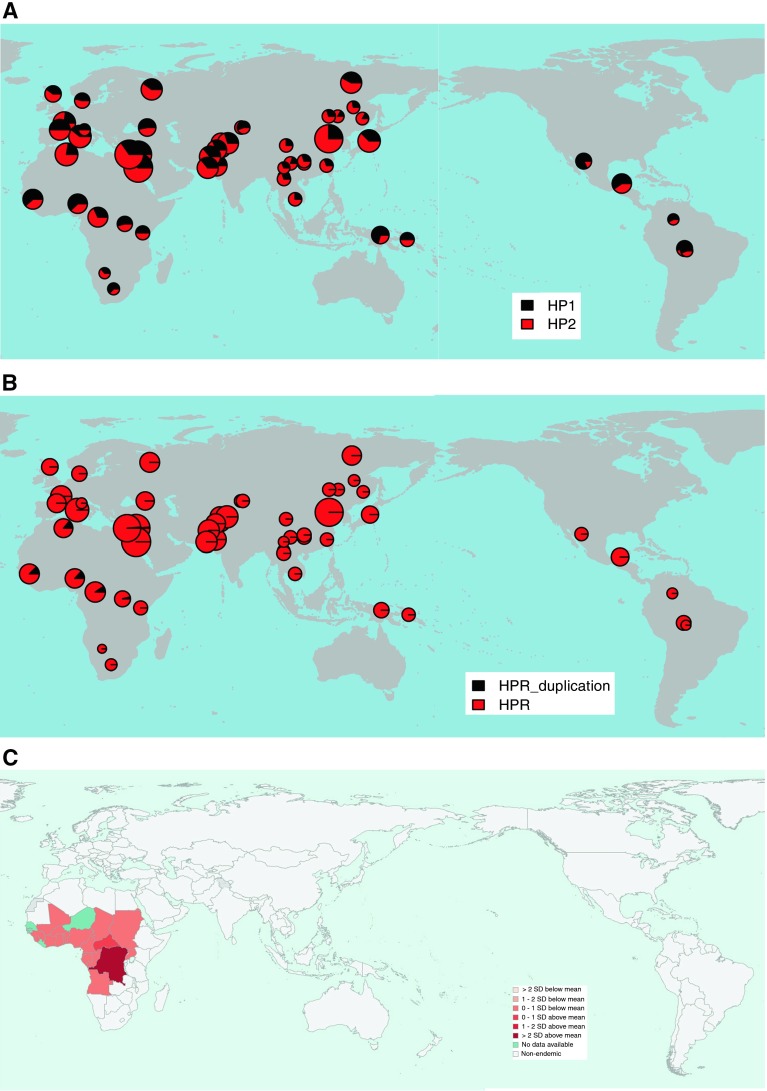

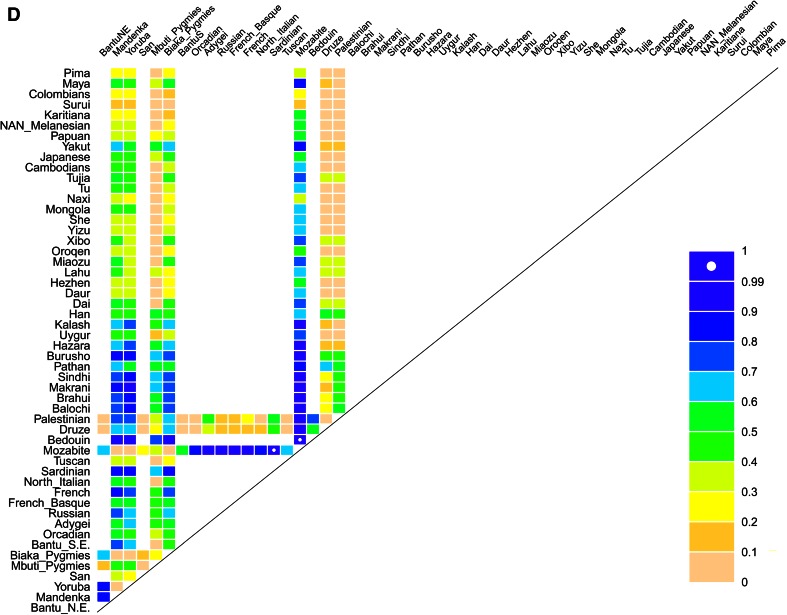
Population distribution of Hp1/2 alleles, *HPR* duplication and *Trypanosoma brucei gambiense.* **a** Distribution of Hp1/2 alleles in the HGDP populations, pies are sized in proportion to sample size. **b** Distribution of *HPR* duplication in the HGDP populations, pies are sized in proportion to sample size. **c** Incidence of human African trypanosomiasis caused by *T. brucei gambiense* in 2010. Data from the World Health Organisation. **d** Pairwise *F*_ST_ statistics for the *HPR* duplication, represented as the percentile of that pairwise *F*_ST_ value in the empirical distribution of values for that population pair, for SNPs with similar minor allele frequencies

The genotype frequencies of the Hp1 allele in the different populations were all in Hardy–Weinberg equilibrium, and the deduced allele frequencies are shown in supplementary table 3 and Fig. 4a.

For *HPR* copy number, we took copy number of 3 as heterozygous duplication, and copy number of four as homozygous *HPR* duplication. The genotype frequencies of the *HPR* duplication allele in the different populations were all in Hardy–Weinberg equilibrium, and the deduced allele frequencies in the HGDP panel populations are shown in supplementary table 3 and Fig. 4b. The *HPR* duplication allele is restricted to Africa, except for two heterozygotes, one Druze and one Palestinian. We found no instances of the duplication in the CEU, JPT and CHB HapMap phase 1 panels, consistent with aCGH data.

As an alternative analysis, we calculated the pairwise *F*_ST_ value between each population for both polymorphisms. For the *HPR* CNV, this is not very informative because the duplication allele is only present in African populations, yet the pairwise *F*_ST_ values for the Mozabite population in particular are unusually high (Fig. 4d; supplementary figure 3a). For the Hp1/2 polymorphism, we can see high *F*_ST_ values for pairwise comparisons involving the Pima and Papuan populations, reflecting a relatively high frequency of Hp1 in those populations (supplementary figure 3b). Because the Hp1/2 polymorphism was one of the first protein polymorphisms identified, there is a considerable amount of population allele frequency data published that has recently been summarised in a review (Carter and Worwood 2007). We took allele frequency data from this review to extend our *F*_ST_ analysis to a total of 122 populations (supplementary Figure 3c). This analysis suggests that the high *F*_ST_ value of the Pima and Papuans is shared with other Native American and Oceanian populations, and forms the only noticeable difference between the population groups.

### Analysis of haplotype context

Initially, haplotypes of the two CNVs and 8 SNPs surrounding the *HP*-*HPR* gene region were analysed (supplementary table 1). Haplotype pairs were called for each sample, with 99.8 % of calls having a posterior probability greater than 0.6, and 83 % of calls having a posterior probability of greater than 0.95. There are four common haplotypes that account for most diversity, with most rare haplotypes being restricted to sub-Saharan Africa. The Hp1 and Hp2 alleles are on several different haplotypes suggesting either recurrent mutation or an old polymorphism that has moved to different haplotype backgrounds by recombination. The analysis also shows that the *HPR* duplication is on one haplotype which included the Hp2 allele (Table 1), confirming the previous observation of association between the two alleles (Maeda et al. 1986).

**Table 1:**
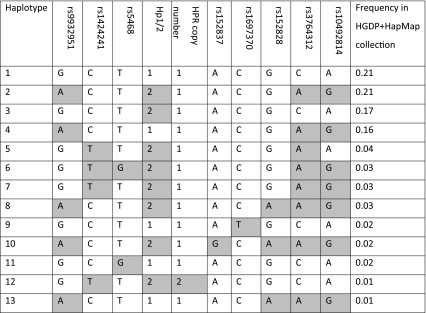
Haplotypes occurring at ≥1 %

Shading indicates derived allele

**Fig. 5:**
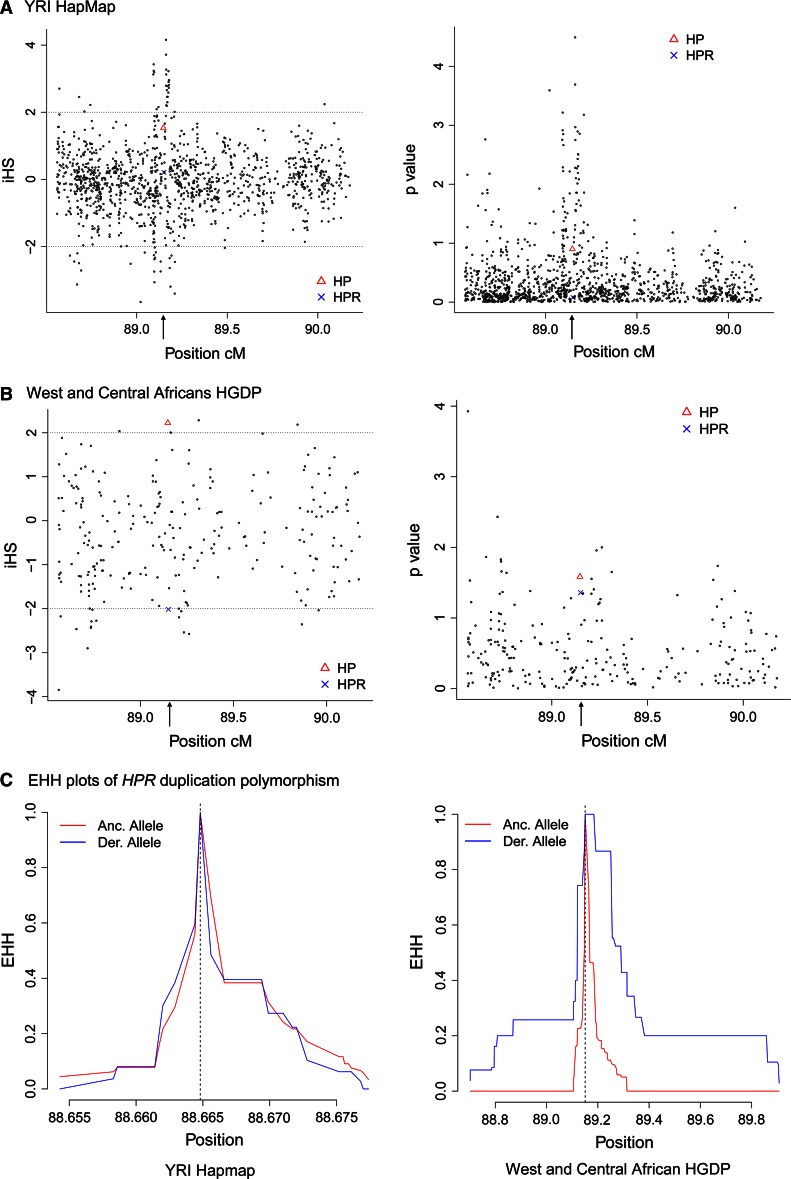
Analysis of signatures of extended haplotypes of the *HP*/*HPR* region. **a** iHS values (left plot) and significance levels of iHS values (right plot) for the YRI population. P values are shown as –log10 (*P* value). **b** iHS values (left plot) and significance levels of iHS values (right plot) for the West and Central African populations. *P* values are shown as –log10 (*P* value). **c** EHH plots for the *HPR* duplication polymorphism for the YRI population (*left*) and West and Central African populations (*right*)

To examine whether haplotypes carrying the *HPR* duplication or the Hp2 allele increased in frequency due to natural selection, we used extended haplotype statistics to detect evidence of a younger-than-expected haplotype, at a particular frequency, associated with the allele of interest. We initially determined extended haplotype homozygosity (EHH) and integrated haplotype score (iHS) for the Hp1/2 and *HPR* duplication polymorphisms using high density SNP data on the YRI trios from the HapMap project. We found no evidence of an extended haplotype for derived alleles at the Hp1/2 and *HPR* duplication polymorphisms, although the *HP* and *HPR* genes appear to be within a region of high iHS scores (Fig. 5a).

To investigate further, we combined the Central and West African populations from the CEPH-HGDP collection together (Mandenka, Yoruba, Mbuti, Biaka), and repeated the analysis. Both Hp1/2 and *HPR* duplication polymorphisms showed nominally significant values for iHS (Table 2; Fig. 5b), the Hp1/2 positive iHS score reflecting an extended haplotype on the ancestral Hp1 allele and the *HPR* duplication negative iHS score reflecting an extended haplotype on the derived duplication allele. Analysis of EHH plots also suggested the existence of an extended haplotype (Fig. 5c). We analysed each population separately to determine whether a stronger signal in one of the populations was responsible for our observation. For the Hp1 allele, there is no convincing evidence of selection in the separate populations. However, for the *HPR* duplication, there is a significant signal of selection for the Yoruba and Biaka populations (Table 2). It should be noted, however, that in all populations stronger signals of selection were present within the 2 Mb region analysed (Table 2), and these overlapping signals are perhaps more likely to account for the hints of extended haplotypes that we see associated with the *HPR* duplication. In addition, the low frequency of the *HPR* duplication places it at the limit of detection of selection by extended haplotype methods.

**Table 2:** Extended haplotype statistics for 2 Mb surrounding the *HP*/*HPR* region

Population	iHS (p) Hp1/Hp2	iHS (p) *HPR* duplication	Strongest iHS signal in region
YRI	1.532 (0.125)	0.208 (0.083)	4.16 (rs7190995)
West/central africa	2.225 (0.026)	−2.016 (0.044)	−3.85 (rs4325560)
Biaka	1.096 (0.080)	−2.009 (0.044)	2.97 (rs1125850)
Yoruba	1.337 (0.181)	−1.995 (0.046)	−3.56 (rs4325560)
Mandenka	1.722 (0.085)	−1.622 (0.105)	−3.56 (rs4325560)
Mbuti	1.956 (0.050)	Frequency <0.05	5.77 (rs7202288)

The breakdown in LD by recombination of a haplotype can be used to estimate the age of an allele on that haplotype independent of frequency. Using this approach, on the combined West and central African data, we estimate the age of the *HPR* duplication to be between 3,400 and 4,200 years old, which is consistent with the adoption of agriculture in West Africa.

### Analysis of pathogen diversity

Figure 4 shows that the *HPR* duplication is in populations that are likely to be exposed, or have been exposed, to *T. brucei gambiense*. Unfortunately, given the small number of analysed populations that have the *HPR* duplication allele and the greatly fluctuating estimates of *T. brucei gambiense* sleeping sickness incidence across the region, a formal correlation analysis with pathogen diversity is likely to yield spurious results, if any. However, it has been previously suggested that malaria prevalence might be responsible for the global variation in Hp1/Hp2 allele frequency. To assess any possible effect of natural selection by pathogen pressure on allele frequency, we correlated the allele frequency of Hp1/2 to a number of pathogen diversity indices, as described previously. The non-parametric partial Mantel test is used, which corrects for the distance from Africa which is the main explanatory variable for allele frequency clines in humans, due to the range expansion out-of-Africa. We found no significant correlation of the Hp2 allele with any pathogen diversity index, including malaria prevalence (data not shown).

### Family-based study of trypanosomiasis and genes encoding TLF-1 components

The lack of power of correlating allele frequencies with pathogen diversity indices above within Africa led us to directly test the hypothesis that the *HPR* duplication allele mediated HAT resistance, presumably through a gene dosage effect, and therefore resistance to HAT might be a possible selective agent acting on the *HPR* duplication allele. We genotyped 135 cases and 218 related individuals for the *HPR* duplication and for two SNPs and an indel in the *APOL1* gene. These three polymorphisms have previously been shown to have undergone natural selection in Yoruba and encode protein variants which show increased ability to lyse trypanosomes (Genovese et al. 2010). Although a relatively small cohort, the family-based approach controls for population stratification, and the sampled individuals are from the Bandundu province of the Democratic Republic of Congo, which has a high prevalence of trypanosomiasis, around 15 %, rising to 70 % in some villages (Ekwanzala et al. 1996). The allele frequency of the *HPR* duplication in unrelated individuals was 0.101, consistent with its distribution in West and Central Africa.

Only one of the polymorphisms shows a significant association by itself (Table 3). We observe, however, that for all four loci the protective allele was undertransmitted to affected individuals (*P* = 0.0125), and that the four loci will mediate variation in trypanosomal lysis via a single functional unit (TLF-1). We reasoned that collapsing all four loci and testing for association jointly was justified, and by doing this we see a significant association of the protective alleles with lack of trypanosomal infection. Following removal of either rs73885319 or rs60910145 locus where the protective alleles are in linkage disequilibrium (supplementary table 4) (Genovese et al. 2010), a significant association remains (Table 3).

**Table 3:** FBAT analysis under an additive model for associations between *HPR* and *APOL1* polymorphisms and HAT

Locus	Protective allele	Allele frequency	Number of informative families	Z value (negative sign indicates undertransmission to HAT cases)	*P* value (1-tailed)
*HPR* copy number	Duplication	0.10	18	−0.745	0.228
*APOL1* rs73885319	G (342 Glycine)	0.15	27	−1.567	0.059
*APOL1* rs60910145	G (348 Methionine)	0.15	21	−1.761	0.039
*APOL1* rs71785313	Deletion	0.09	11	−1.604	0.055
All combined	All protective alleles	–	–	−2.240	0.0125
Combined, without rs73885319	All protective alleles	–	–	−1.896	0.0289
Combined, without rs60910145	All protective alleles	–	–	−1.719	0.0428
Combined, without HPR	All protective alleles	–	–	−2.131	0.0165

## Discussion

In this study we characterise the *HPR* duplication, which has been observed previously only in African-Americans, and, based on its allele frequency distribution, confirm its likely origin in West Africa. The original report describing the *HPR* duplication also described individuals with higher copy number, up to 6 copies of *HPR* on a single chromosome, characterised by Southern blot. We found no evidence of higher *HPR* copy numbers beyond a simple duplication, so we consider that these high-copy number *HPR* chromosomes are very rare in the population. It should be noted that the original study selected some individuals on the basis of unusual haemoglobin phenotype, so, given that Hpr binds haemoglobin, it is possible that this enriched for unusual *HPR* genotypes. We show that the *HPR* duplication is on one haplotype and is therefore likely to have occurred once, and we confirm the original study that the *HPR* duplication occurred on an Hp2 allelic background (Maeda et al. 1986).

The distribution of *HPR* focusing on Central and West Africa supports a hypothesis where increased levels of *HPR* (a component of TLF-1), and hence higher *HPR* copy number alleles, are selected for because of improved resistance to HAT. We examined the surrounding genomic region for signatures of natural selection based on extended haplotype tests. These detect recent hard selective sweeps, and there is previously published evidence suggesting that such a selective sweep acted on alleles at the *APOL1* gene that show resistance against *T. brucei* (Genovese et al. 2010; Ko et al. 2013). The evidence for a similar sweep acting on the *HPR* duplication is equivocal, with the observed extended haplotype better explained by stronger selective sweeps acting on different SNP alleles within the 2 Mb region analysed. By itself, there is no evidence that the *HPR* duplication is associated with protection against human African trypanosomiasis in an area of Central Africa where the *T. b. gambiense* parasite is highly endemic, and causes repeated epidemics of sleeping sickness. However, taken together with alleles at the *APOL1* gene, the data are consistent with a role of the *HPR* locus in increased resistance to *T. b. gambiense* sleeping sickness as a possible selective agent by increasing the effectiveness of TLF-1. The association should be treated with caution, as the study is rather underpowered to detect small effects of variants with allele frequencies <0.2, and ideally should be confirmed in a larger cohort, if such a cohort was available. We also do not test for a gene dosage effect of the *HPR* duplication, but this is not straightforward given the rarity of the duplication allele and the similarity at the protein level between haptoglobin-related protein and haptoglobin, the latter also being present at much higher levels than the former in serum from healthy individuals (Muranjan et al. 1998). Taken together, the data described in this study suggest that, in vivo, the HAT-protective allelic variants of *APOL1* and *HPR* help the host to overcome the reduced affinity of the haptoglobin receptor for TLF-1 that characterises *T. b. gambiense*, and this should be tested experimentally. The HP/HPR region varies in copy number in rhesus macaques (Perry et al. 2008), and given that trypanosomes naturally occur in macaques, this might be an alternative model system for further analysis.

The observation of *HPR* duplication alleles at significant frequency in the Mozabite (Berber) population of Algeria is perhaps surprising, as they are a non-sub-Saharan population, often grouped with Middle Eastern populations, and Algeria is not a country with endemic *Trypanosoma*. However, pollen analysis shows that North Africa was more lush 6,000 years ago compared to the arid conditions seen today, and therefore may have been within the range of the tsetse fly, the vector of *T. brucei* (Jolly et al. 2008; Steverding 2008). Presence of trypanosomiasis, at least in animals, was recorded by the ancient Egyptians 4,000 years ago in an area now free of the disease and the vector, so it is possible that the observation of the *HPR* duplication in North Africa and the Middle East is a result of selective events in the past when trypanosomiasis may have been endemic. Alternatively, it is known that the Mozabite were nomadic, and roamed as far south as the Niger and Senegal rivers in West Africa, so they may have inherited the *HPR* duplication from populations to the south in tropical endemic areas. The dating of the *HPR* duplication allele between 3,400 and 4,200 years ago suggests that it originated soon after the development of agriculture in West Africa, possibly after the drying of the Sahara region and the consequent southward move of the northern limit of the tsetse fly, the vector for trypanosomiasis.

It has previously been suggested that the distribution of the Hp2 allele has been driven by malaria selection pressure. Our data do not support this, because we did not find any correlation between the Hp2 allele and a number of pathogen diversity indices, including two malaria prevalence indices and a protozoan diversity index, of which a large proportion is due to *Plasmodium falciparum* and *P. vivax*. There is a caveat in our data in that our malarial prevalence estimates are on a country-by-country basis for 21 countries, and of course reflect current prevalence levels rather than prevalence levels in the past that may have given rise to the different allele frequencies seen today. Nevertheless, alleles of different well-known genes that are likely to have undergone selection by malaria have been identified using this approach, such as *GYPC* (glycophorin C)*, ABO* (ABO blood group), and *SLC4A1* (erythrocyte membrane protein band). A recent study also suggests that an uncommon haplotype carrying the Hp2 allele shows some evidence of long extended haplotypes characteristic of recent natural selection, but in light of our data this seems to be a signal of selection at the *HPR* duplication or another nearby allele (Rodriguez et al. 2012).

There are several examples of CNV mediating different susceptibilities to infectious diseases (Hardwick et al. 2012; Mockenhaupt et al. 2004; Pelak et al. 2011). Despite the fact that infectious disease is likely to have been, and remains, a strong agent of natural selection on humans, detection of signatures of selection at copy number variable regions remains difficult, and typically relies on the identification of unusually high genetic differentiation between populations or continents (Hardwick et al. 2011; Iskow et al. 2012; Perry et al. 2007). Extended haplotype tests for selection can be used only when a particular copy number allele occurs on one haplotype. Here we investigate a possible example of natural selection for infectious disease resistance increasing the frequency of a copy number variant. The functional basis for this selection is well supported, and the other component of TLF-1, *APOL1*, also shows a similar sign of selection in west Africa (Genovese et al. 2010). However, our data are equivocal on the evidence for a selective advantage of the *HPR* duplication. This may be a real observation, or it may be that our analyses are underpowered because of small sample sizes, particularly in the context of a duplication allele frequency between 0.1 and 0.15. Further data from west and central African populations are required to fully characterise the patterns of selection in this genomic region, and a larger epidemiological study of HAT would also be an important future research avenue. HAT has had a profound impact on human and domestic animal evolution, and understanding its effect on genomes remains an important goal.

## Electronic supplementary material

Below is the link to the electronic supplementary material.

Supplementary material 1 (DOC 1.2 MB)
