# Supplementary material for: Haptoglobin (HP) and Haptoglobin-related protein (HPR) copy number variation, natural selection, and trypanosomiasis
Source: Hum Genet. 2013 Sep 5;133(1):69–83. doi: 10.1007/s00439-013-1352-x (PMC3898332; doi:10.1007/s00439-013-1352-x)
Supplement: Supplementary file 1 — Supplementary material 1 (DOC 1.2 MB) [file 439_2013_1352_MOESM1_ESM.doc]

**Supplementary Material for Hardwick et al.**

**Haptoglobin (*HP*) and Haptoglobin-related protein (*HPR*) copy number variation, natural selection and trypanosomiasis**

**Contents**

Supplementary table 1 PCR Primers for assays 2

Supplementary table 2 Control samples and genotypes 2

Supplementary table 3 Estimated allele frequencies in HGDP populations 3

Supplementary table 4 Linkage disequilibrium values (Dʹ) between different

polymorphisms in the Yansi 4

Supplementary figure 1 Genotyping the Hp1/2 polymorphism 5

Supplementary figure 2 Analysis of array CGH signal in a *HPR* duplication heterozygote 6

Supplementary figure 3 Plots of pairwise F_ST_ 7

**Supplementary Table 1 PCR Primers for assays**

| **Assay** | **Primer Name** | **Primer Sequence** (5’-3’) | **Predicted DNA Fragment Size (bp)** |
| --- | --- | --- | --- |
| *HPR* PRT Assay 1 | HP_HPR_L-F | [HEX]GAGGGAGGTTTCTCTTTCCTG | 169  172 |
|  | HP_HPR_L-R | CTTCTGAGCATACCAAGCTTCC |  |
| *HPR* PRT Assay 2 | HP_PRT_F | [6FAM]GCCTTGTTGCAGTGGCCTC | 146, 145,  164, 168 |
|  | HP_PRT_R | ACATTTCTTACCTTGGTCTG |  |
| *HP* Genotyping Assay | Hp-A | GAGGGGAGCTTGCCTTTCCATTG | 3481 (Hp2)  1757 (Hp1) |
|  | Hp-B | GAGATTTTTGAGCCCTGGCTGGT |  |
|  | Hp-C | CCTGCCTCGTATTAACTGCACCAT | 349 (Hp2) |
|  | Hp-D | CCGAGTGCTCCACATAGCCATGT |  |
| *APOL1* Genotyping Assay | APOL1F | [HEX]GAGCAGAGGAGTCAAGCTCAC | 212 |
|  | APOL1R | GTCACAGTTCTTGGTCCGC |  |

**Supplementary Table 2 Control samples and genotypes**

| Sample | Population | HP1/2 genotype | HPR copy number | HPR copy number validation |
| --- | --- | --- | --- | --- |
| NA18572 | YRI | 2-2 | 2 | aCGH |
| NA18507 | YRI | 2-1 | 2 | Fiber-FISH, aCGH |
| NA18854 | YRI | 1-1 | 2 | aCGH |
| NA18862 | YRI | 2-2 | 3 | aCGH |
| NA19240 | YRI | 2-1 | 3 | Fiber-FISH, aCGH |
| NA18503 | YRI | 2-2 | 4 | Fiber-FISH, aCGH |
| NA19221 | YRI | 2-2 | 4 | Fiber-FISH, aCGH |

**Supplementary table 3 Estimated allele frequencies in HGDP populations**

| Population | Hp1  frequency | *HPR*_dup  frequency |
| --- | --- | --- |
| Adygei | 0.53 | 0.00 |
| Balochi | 0.31 | 0.00 |
| Bantu_N.E. | 0.50 | 0.00 |
| Bantu_S.W. | 0.63 | 0.00 |
| Bedouin | 0.38 | 0.00 |
| Biaka_Pygmy | 0.32 | 0.10 |
| Brahui | 0.36 | 0.00 |
| Burusho | 0.30 | 0.00 |
| Cambodian | 0.25 | 0.00 |
| Colombian | 0.57 | 0.00 |
| Dai | 0.30 | 0.00 |
| Daur | 0.17 | 0.00 |
| Druze | 0.27 | 0.01 |
| French | 0.23 | 0.00 |
| French_Basque | 0.50 | 0.00 |
| Han | 0.25 | 0.00 |
| Hazara | 0.32 | 0.00 |
| Hezhen | 0.17 | 0.00 |
| Japanese | 0.38 | 0.00 |
| Kalash | 0.24 | 0.00 |
| Karitiana | 0.54 | 0.00 |
| Lahu | 0.19 | 0.00 |
| Makrani | 0.36 | 0.00 |
| Mandenka | 0.61 | 0.13 |
| Maya | 0.60 | 0.00 |
| Mbuti_Pygmy | 0.54 | 0.04 |
| Miaozu | 0.20 | 0.00 |
| Mongola | 0.30 | 0.00 |
| Mozabite | 0.36 | 0.14 |
| NAN_Melanesian | 0.50 | 0.00 |
| Naxi | 0.31 | 0.00 |
| North_Italian | 0.46 | 0.00 |
| Orcadian | 0.40 | 0.00 |
| Oroqen | 0.28 | 0.00 |
| Palestinian | 0.35 | 0.01 |
| Papuan | 0.71 | 0.00 |
| Pathan | 0.23 | 0.00 |
| Pima | 0.82 | 0.00 |
| Russian | 0.40 | 0.00 |
| San | 0.36 | 0.00 |
| Sardinian | 0.39 | 0.00 |
| She | 0.30 | 0.00 |
| Sindhi | 0.27 | 0.00 |
| Surui | 0.56 | 0.00 |
| Tu | 0.25 | 0.00 |
| Tujia | 0.30 | 0.00 |
| Tuscan | 0.50 | 0.00 |
| Uygur | 0.30 | 0.00 |
| Xibo | 0.56 | 0.00 |
| Yakut | 0.42 | 0.00 |
| Yizu | 0.20 | 0.00 |
| Yoruba | 0.62 | 0.13 |

**Supplementary table 4 Linkage disequilibrium values (Dʹ) between different polymorphisms in the Yansi**

| Locus | *HPR* copy number | *APOL1* rs73885319 | *APOL1* rs60910145 | *APOL1* rs71785313 |
| --- | --- | --- | --- | --- |
| *HPR* copy number | - | - | - | - |
| *APOL1* rs73885319 | 0.17 | - | - | - |
| *APOL1* rs60910145 | 0.17 | 1.00 | - | - |
| *APOL1* rs71785313 | 0.02 | 0.35 | 0.67 | - |

**Supplementary figure 1 Genotyping the Hp1/2 polymorphism**

Ethidium bromide-stained agarose gel showing PCR products used to genotype the Hp1/2 polymorphism. Lane 4 contains the negative control. Lanes 1 and 5 are from individual A (NA18572), Lanes 2 and 6 are from individual B (NA18507) and Lanes 3 and 7 are from individual C (NA18854). Using primers C and D occasionally a weak band generated by mis-priming from the Hp1 allele was observed (as seen here, but, if present, the intensity was always much less than true positives.


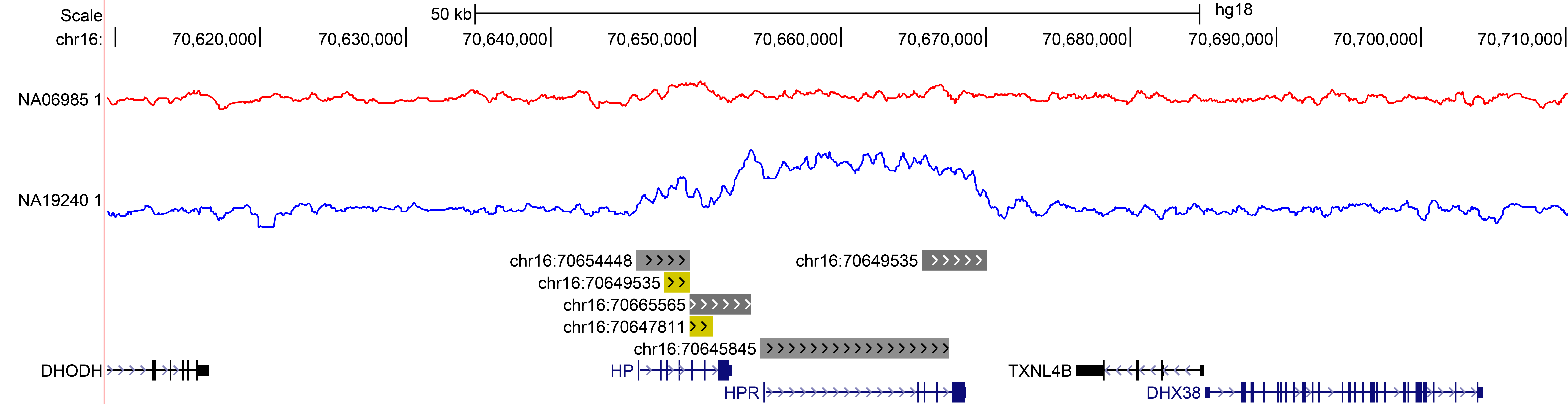


**Supplementary figure 2 Analysis of array CGH signal in a *HPR* duplication heterozygote**

A sliding window average of the normalised log2 ratios was calculated, with a window size of 10 probes, for 100kb flanking the *HP* gene on two HapMap samples: NA19240, carrying a likely *HPR* duplication, and NA06985 carrying two copies of the non-duplicated allele. Data was plotted using Genome Graphs facility of the UCSC Genome Browser, assembly hg18. Segmental duplication and UCSC Gene tracks are also shown.

**
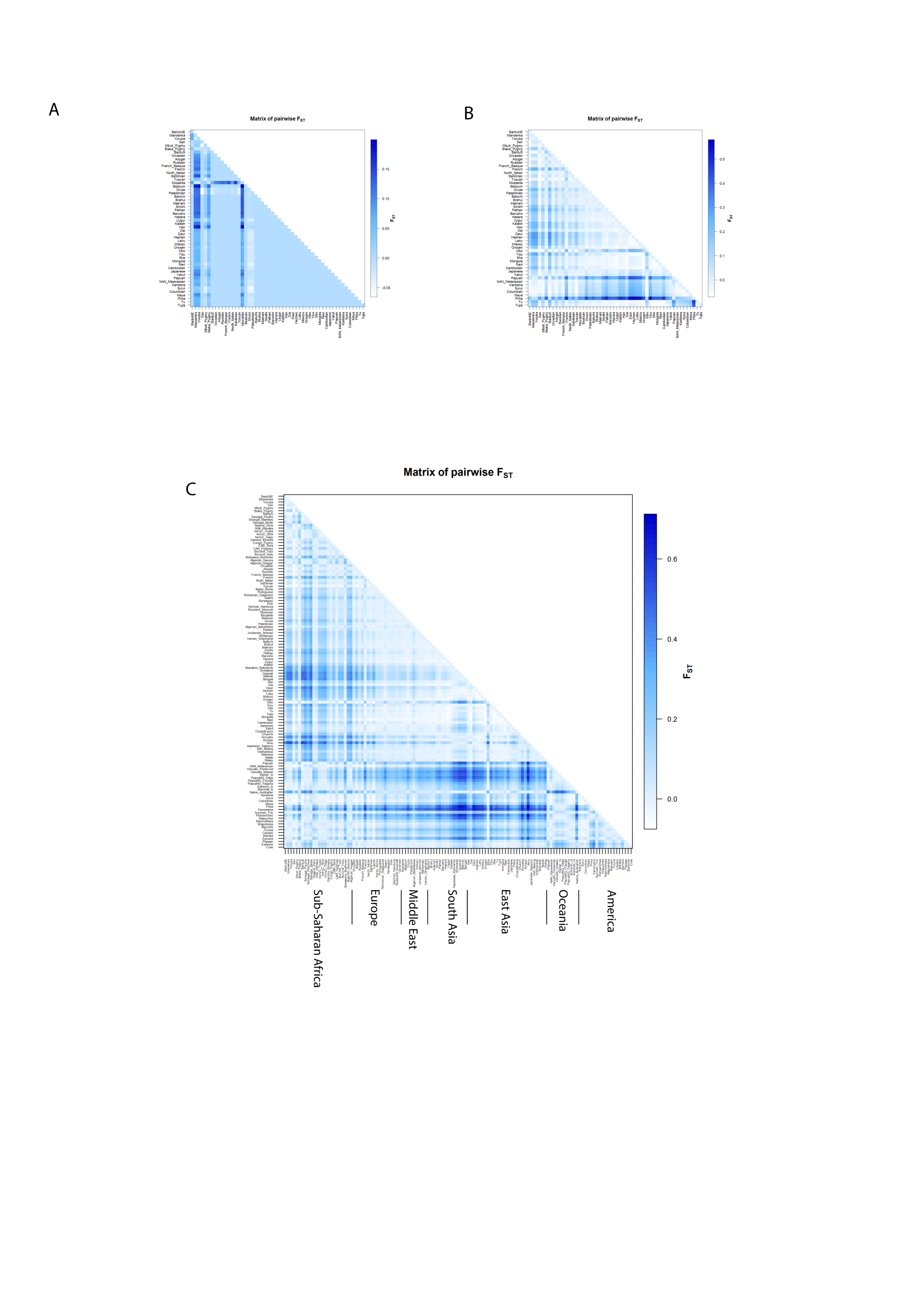
**

**Supplementary figure 3 Plots of pairwise F_ST_**

1. *HPR* duplication
2. Hp1/2
3. Hp1/2 for an extended dataset
